# Pediatric Dental Management of a Patient With Infantile Osteopetrosis in Remission: A Clinical Case Report

**DOI:** 10.1155/crid/7684477

**Published:** 2026-04-15

**Authors:** Maria Amalia Cruz-Morera, Katherine Molina-Chaves, Adrian Gomez-Fernandez

**Affiliations:** ^1^ Faculty of Dentistry, University of Costa Rica, San José, Costa Rica, ucr.ac.cr

**Keywords:** dental anomalies, enamel hypoplasia, oligodontia, osteopetrosis, pediatric dentistry

## Abstract

**Background:**

Osteopetrosis is a rare genetic disorder characterized by increased bone density due to defective osteoclast function. Its clinical presentation varies according to subtype, and oral manifestations are frequent, potentially serving as early indicators of the disease. These findings place the pediatric dentist in a key role for early recognition and safe dental management.

**Case:**

This case report describes the comprehensive dental management of a 7‐year‐old girl with a history of infantile osteopetrosis treated with hematopoietic stem cell transplantation during early infancy. The patient presented with oligodontia, generalized enamel hypoplasia and hypomineralization, dental hypersensitivity, and altered dental morphology. Management focused on prevention‐centered care, including professional prophylaxis, topical fluoride therapy (5% sodium fluoride varnish), home fluoridated toothpaste and mouth rinse use, pit and fissure sealants, selective minimally traumatic extractions with antibiotic prophylaxis, and interceptive orthodontic treatment using slow maxillary expansion with a Bertoni appliance. Monthly follow‐up visits were conducted over a 12‐month period.

**Results:**

Enamel defects remained stable, dental hypersensitivity decreased, no new carious lesions developed, and no infectious or osteonecrotic complications were observed. Slow maxillary expansion improved arch coordination without adverse effects.

**Conclusion:**

A conservative, prevention‐oriented, and multidisciplinary dental approach can be safely implemented in pediatric patients with osteopetrosis. Early identification, minimally invasive procedures, and close follow‐up are essential to reduce complications and improve oral health outcomes and quality of life.

## 1. Introduction

Infantile osteopetrosis is a rare inherited bone disorder characterized by abnormally increased bone density resulting from impaired bone remodeling. This condition is caused by defective or absent osteoclast function, which leads to the accumulation of dense yet brittle bone with a characteristic glass‐like appearance on radiographic imaging. Osteopetrosis encompasses a heterogeneous group of clinical entities, most commonly presenting as a benign autosomal dominant form or a severe autosomal recessive form. Its etiology involves mutations in genes such as *CLCN7* and *TCIRG1*, which play critical roles in osteoclast differentiation and function [1]. Depending on the underlying genetic mutation, affected patients may develop a wide range of clinical manifestations, including recurrent fractures, anemia, cranial nerve compression, and severe immunodeficiency [2].

The global prevalence of osteopetrosis is estimated to range from 1 in 100,000 to 1 in 500,000 live births, with pathogenic variants identified in at least 10 genes accounting for the diverse phenotypic spectrum of the disease [3]. The condition affects both sexes, although clinical severity is primarily determined by the underlying genetic mutation rather than biological sex. In contrast, Costa Rica has reported a considerably higher incidence, estimated at ~3.4 cases per 100,000 live births, compared with international figures of around 2 cases per 250,000. This increased national incidence has been largely attributed to hereditary factors, including consanguinity in certain populations, and is associated with a higher prevalence of the malignant autosomal recessive form of the disease [4].

Clinically, osteopetrosis is classified into three main forms: infantile malignant, intermediate, and adult, each differing in severity and prognosis. The disease may present during early infancy and is characterized by a broad spectrum of systemic manifestations, including growth disturbances such as short stature and macrocephaly, anemia, hepatosplenomegaly, and neurological complications secondary to cranial nerve compression, which may lead to visual or auditory impairment. Craniofacial and oral manifestations are frequent and may include rampant caries, enamel hypoplasia, delayed or failed tooth eruption, malformed roots, congenitally missing teeth, and mandibular osteomyelitis, particularly following dental extractions or odontogenic infections [5].

The differential diagnosis includes other conditions that affect skeletal and dental development, such as hereditary bone dysplasias and metabolic disorders, which can be distinguished through a combination of genetic, radiographic, and laboratory investigations. From a dental perspective, oligodontia associated with osteopetrosis is frequently accompanied by delayed or failed tooth eruption, conical crowns, short or absent roots, and enamel hypoplasia. This pattern differs from conditions such as ectodermal dysplasia, in which oligodontia is associated with ectodermal systemic features and occurs in the absence of increased bone density [6].

Radiographically, osteopetrosis is characterized by generalized osteosclerosis, most prominently affecting the vertebrae, pelvis, and metaphyses of long bones, where classic findings such as the “bone–within–bone” appearance and the “sandwich vertebrae” may be observed. In the maxillofacial region, imaging may reveal alterations in mandibular architecture, areas of osteonecrosis associated with odontogenic infections, reduced medullary bone spaces, and narrowing of optic nerve canals [7].

In selected cases, bone biopsy may be performed to aid in the differentiation of disease subtypes, revealing osteoclasts that are normal or increased in number but functionally impaired. This dysfunction prevents effective bone resorption, leading to the accumulation of non‐remodeled primary bone and alterations in bone matrix composition. Consequently, the bone becomes dense, brittle, and poorly vascularized, with reduced functional marrow spaces that compromise hematopoiesis. Nevertheless, the role of bone biopsy is limited, as genetic and radiographic studies are generally sufficient for diagnosis and disease classification [8].

Therapeutic management depends on disease severity and clinical subtype. In severe forms, such as malignant infantile osteopetrosis, hematopoietic stem cell transplantation remains the treatment of choice, as it enables the restoration of functional osteoclast activity. In milder forms, management is primarily supportive and may include calcium and vitamin D supplementation, as well as antibiotic therapy in the presence of complications such as osteomyelitis. A multidisciplinary approach involving hematology, neurology, and dentistry is essential to minimize complications and to improve patient outcomes and quality of life [8].

From a dental standpoint, management of patients with osteopetrosis requires special caution. The increased bone density and reduced vascularity characteristic of this condition compromise normal bone remodeling and healing capacity, predisposing patients to postoperative complications such as delayed healing, osteomyelitis, and osteonecrosis, particularly after extractions or invasive procedures. Consequently, minimally traumatic approaches, strict infection control measures, and prevention‐centered strategies are strongly recommended. In addition, orthodontic interventions may present biomechanical challenges due to the sclerotic nature of the bone, requiring gradual and carefully controlled forces to avoid tissue damage. These considerations highlight the need for individualized, conservative, and multidisciplinary dental management protocols [9, 10].

The aim of this case report is to describe the comprehensive dental management of a pediatric patient with infantile osteopetrosis in remission, highlighting the associated clinical challenges and the importance of interdisciplinary preventive care.

## 2. Case Report

A 7‐year‐old female patient was referred to the pediatric dentistry clinic by the pediatric hematology service for comprehensive oral evaluation and planning of restorative and orthodontic management. Written informed consent for publication of this case and accompanying clinical images was obtained from the patient’s parent, and assent was obtained from the patient prior to the preparation of this report.

The patient had a medical history of severe infantile osteopetrosis, diagnosed at 4 months of age in 2016. The condition had been successfully treated with hematopoietic stem cell transplantation, with her mother serving as the donor, resulting in sustained clinical remission.

At the time of dental assessment, the patient was under regular follow‐up by the pediatric hematology service, with no evidence of disease recurrence and no ongoing immunosuppressive therapy. Growth parameters, including height and weight, were within normal percentiles for her age, and no systemic complications were reported.

Following hematopoietic stem cell transplantation, several dental sequelae became evident, including oligodontia and multiple developmental enamel defects. Intraoral clinical examination revealed generalized enamel hypoplasia and hypomineralization affecting both anterior and posterior teeth, dental hypersensitivity, and alterations in crown and root morphology. Oral hygiene was initially fair, with localized plaque accumulation but no active cavitated caries lesions. Initial occlusal photographs of the maxillary and mandibular arches documented altered dental morphology, enamel defects, and space discrepancies associated with oligodontia.

Panoramic radiographs obtained at two different time points, April 2024 (initial assessment) and November 2024 (follow‐up), demonstrated congenital absence of multiple teeth and persistent alterations in dental development, including abnormalities in root formation, without evidence of progressive pathological changes (Figure 1). No palpable cervical lymphadenopathy was detected, and extraoral examination revealed no visible abnormalities.

Figure 1Panoramic radiographs obtained at two different time points showing multiple congenitally missing teeth and persistent alterations in dental development in a pediatric patient with a history of infantile osteopetrosis: (A) April 2024; (B) November 2024.

**Figure:**
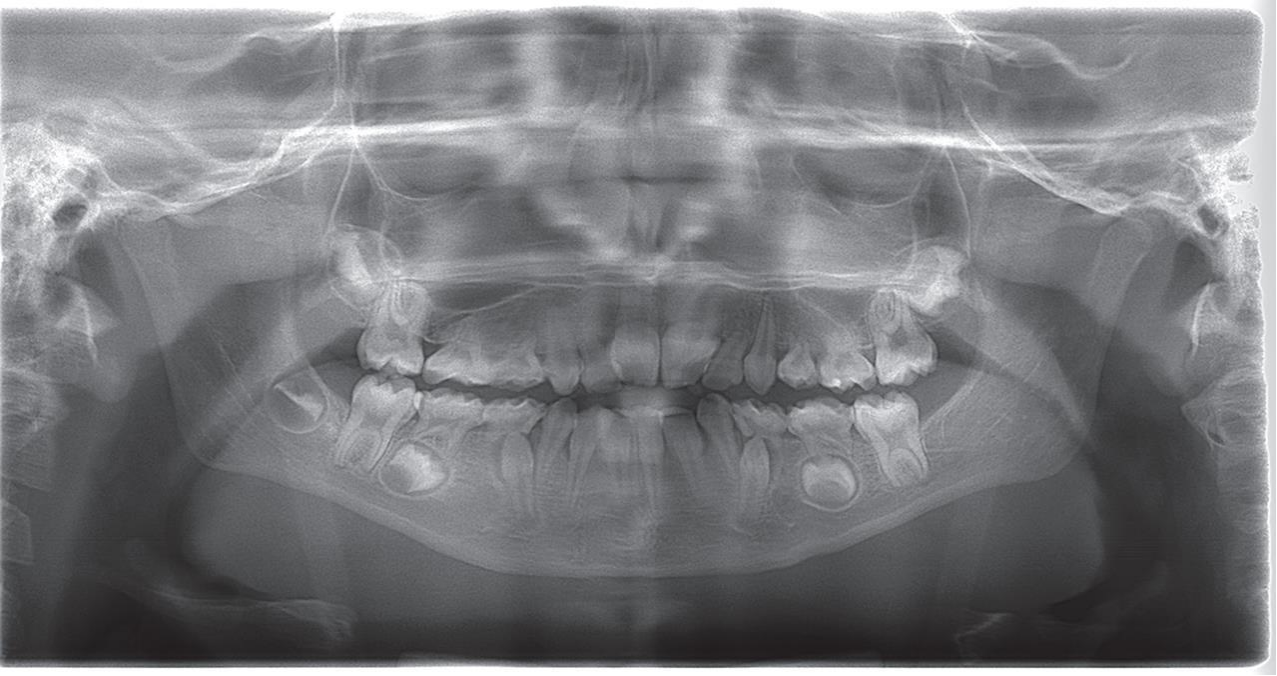
(A)

**Figure:**
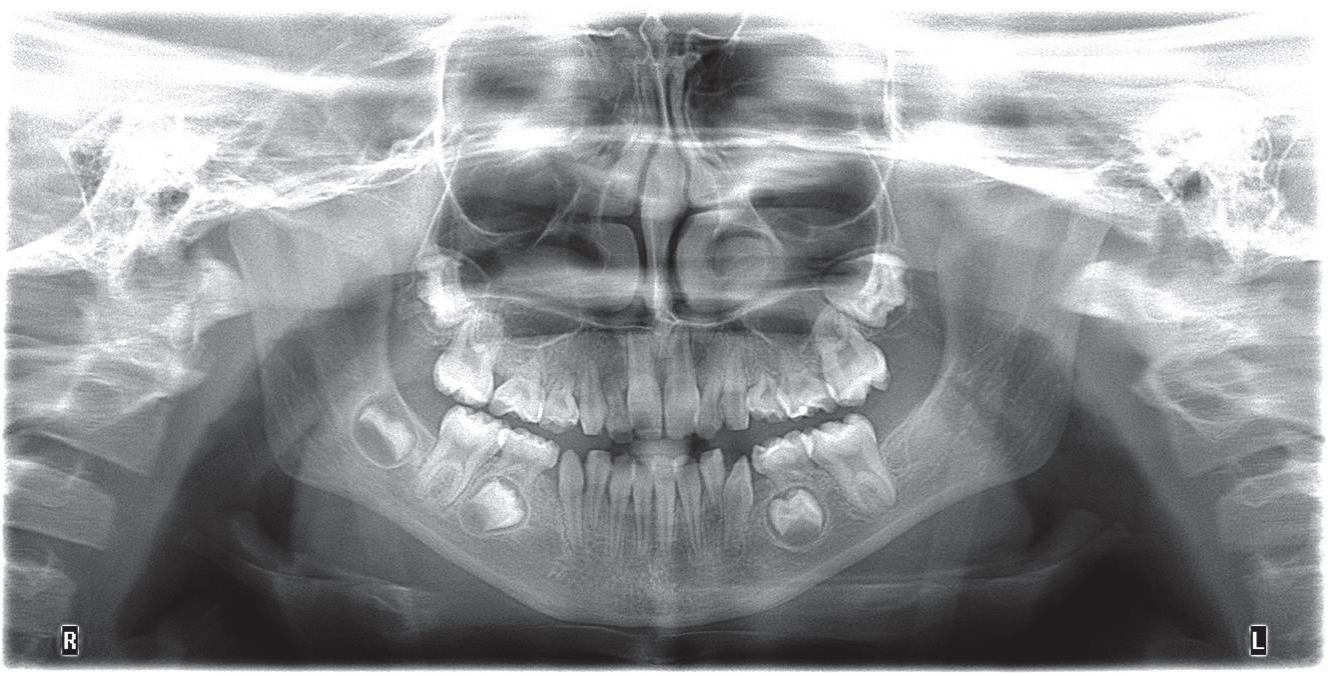
(B)

Based on the diagnosis of dental sequelae associated with osteopetrosis—namely oligodontia, enamel hypoplasia, and hypomineralization—a comprehensive and individualized treatment plan was established (Figure 2). The primary objectives were to prevent caries development, reduce hypersensitivity, avoid infectious complications, and guide craniofacial growth through conservative orthodontic intervention.

Figure 2Frontal and lateral intraoral views showing enamel hypoplasia and hypomineralization affecting multiple tooth surfaces: (A) frontal view; (B) right lateral view; (C) left lateral view.

**Figure:**
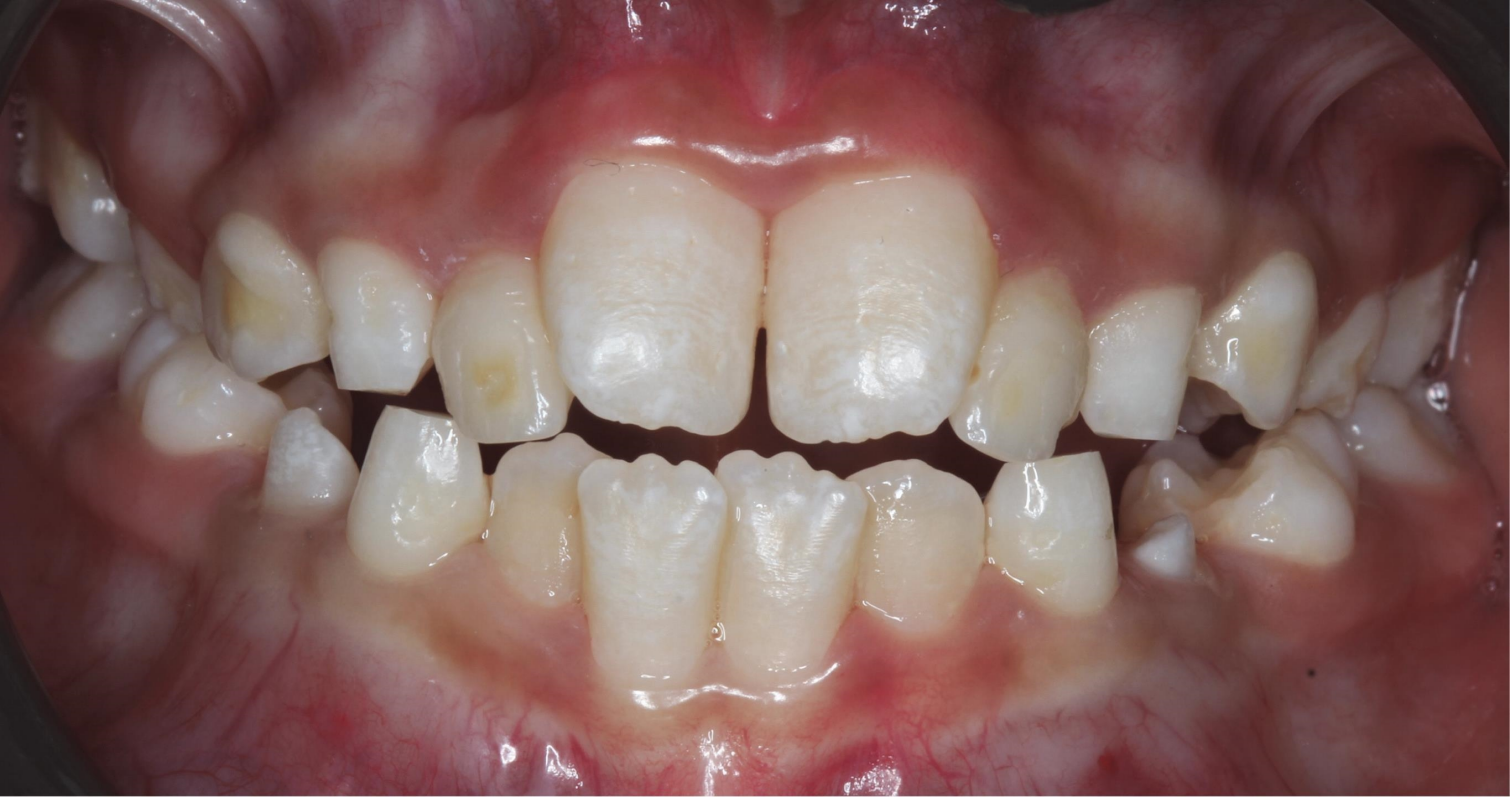
(A)

**Figure:**
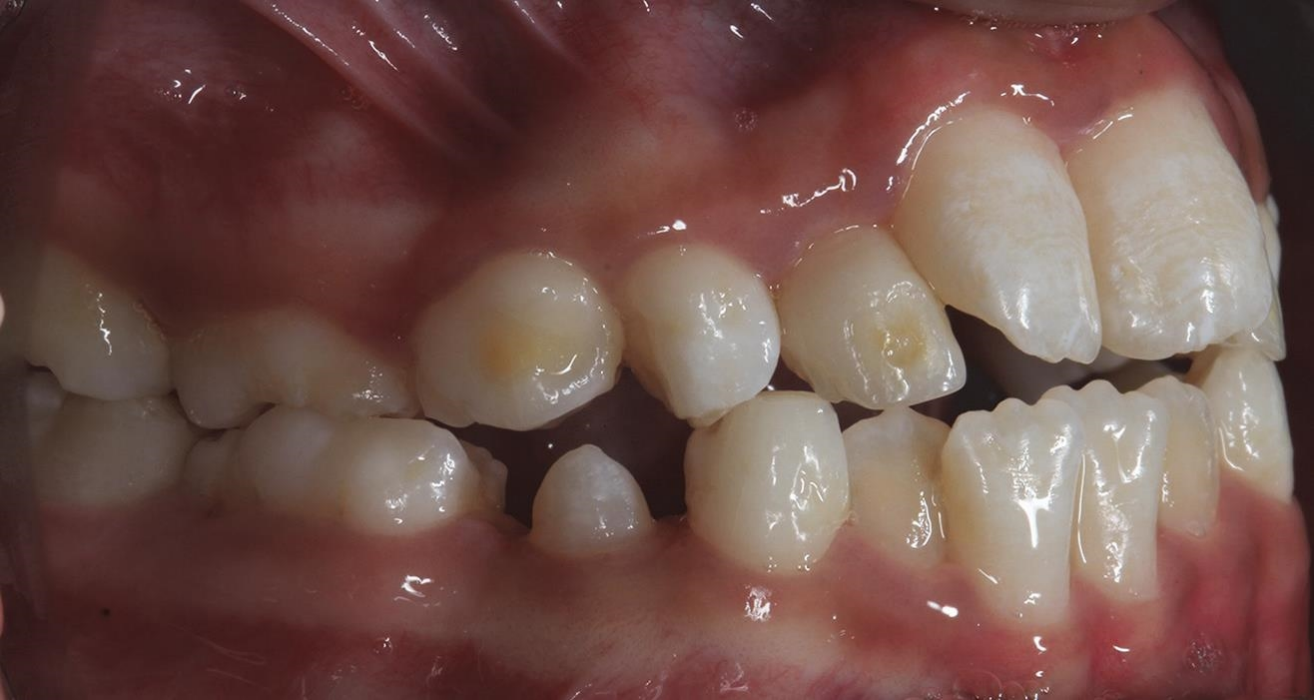
(B)

**Figure:**
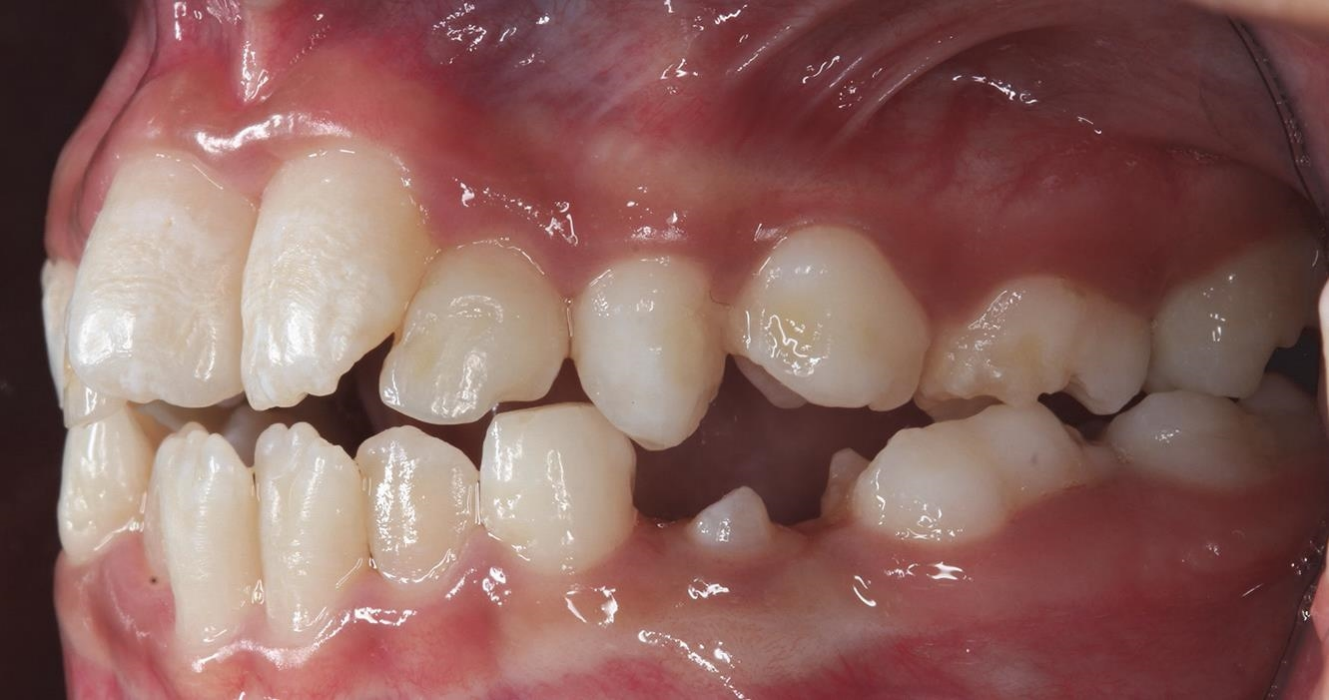
(C)

Preventive management included professional dental prophylaxis at each visit and topical application of 5% sodium fluoride varnish (22,600 ppm fluoride) every 3 months. At home, the patient was instructed to brush twice daily with 1450 ppm fluoridated toothpaste and to use a weekly 0.2% sodium fluoride mouth rinse under parental supervision. Dietary counseling focused on reducing the frequency of fermentable carbohydrate intake. Pit and fissure sealants were placed on susceptible permanent molars, and desensitizing agents were applied to hypersensitive teeth.

Selective extractions were limited to retained primary molars presenting grade III mobility and poor prognosis. Due to the increased risk of osteomyelitis associated with osteopetrosis, all procedures were performed using minimally traumatic, flapless techniques under local anesthesia, with careful preservation of surrounding bone. Strict aseptic protocols were followed, and short‐term antibiotic prophylaxis with amoxicillin (50 mg/kg/day for 5 days) was prescribed. This decision was made in consultation with the patient’s hematology team due to the increased risk of osteomyelitis reported in patients with osteopetrosis undergoing dental extractions. Healing was monitored weekly, and no postoperative complications were observed.

Interceptive orthodontic treatment was indicated due to transverse maxillary constriction and reduced arch perimeter secondary to oligodontia and altered eruption patterns. Slow maxillary expansion using a Bertoni appliance was selected to deliver gradual and controlled orthopedic forces, minimizing the risk of bone complications in the context of increased bone density. Activation was performed at a rate of 0.25 mm per week, allowing physiological adaptation of the surrounding tissues. During the orthodontic phase, monthly monitoring included clinical evaluation of tooth mobility, periodontal tissue condition, eruption pattern, and patient‐reported symptoms to detect potential complications associated with orthodontic forces in sclerotic bone. Periodic adjustments were performed during the monthly follow‐up visits (Figure 3).

Figure 3Occlusal view of a Bertoni appliance used for slow maxillary expansion as part of interceptive orthodontic management in a pediatric patient with altered bone metabolism: (A) intraoral occlusal view; (B) extraoral view of the appliance.

**Figure:**
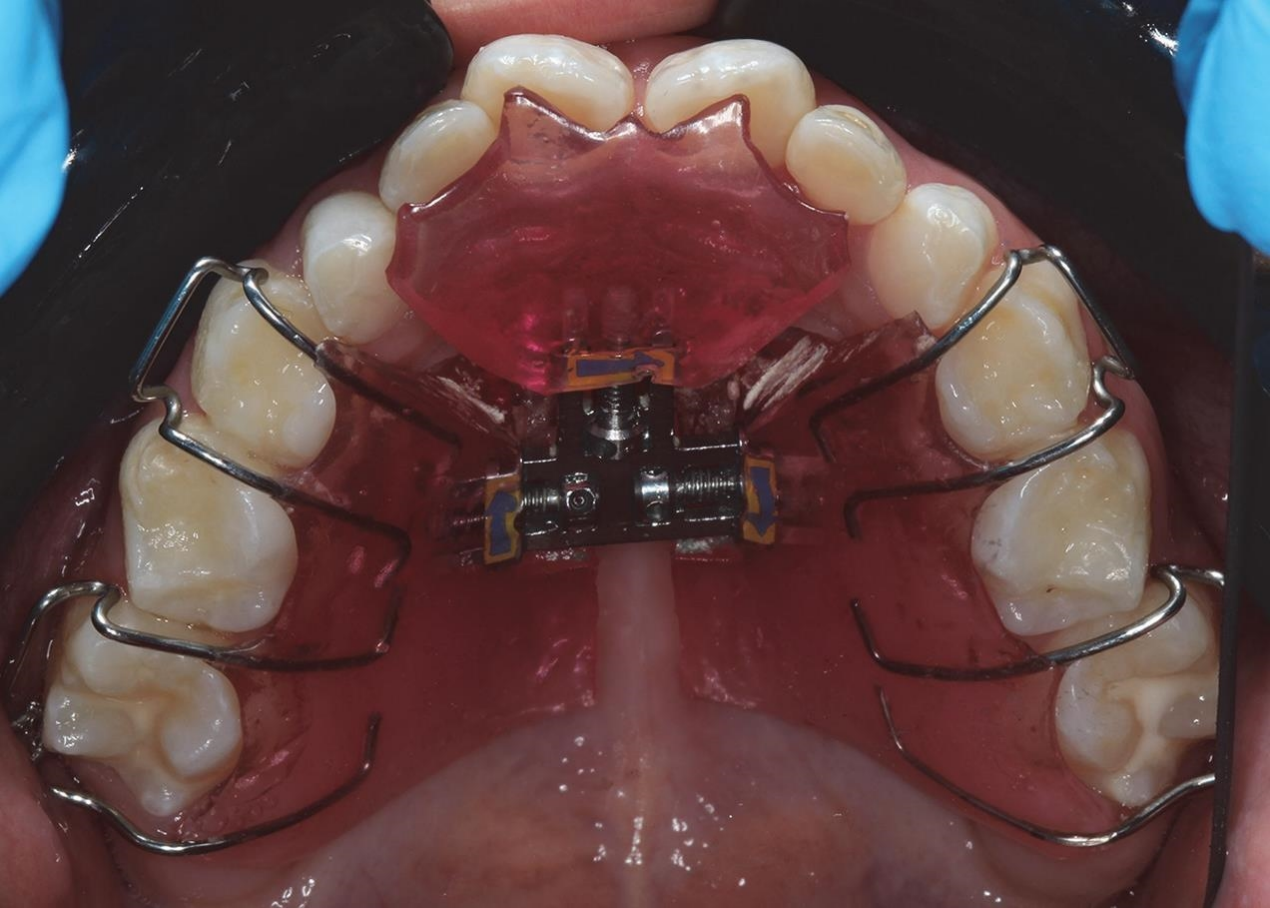
(A)

**Figure:**
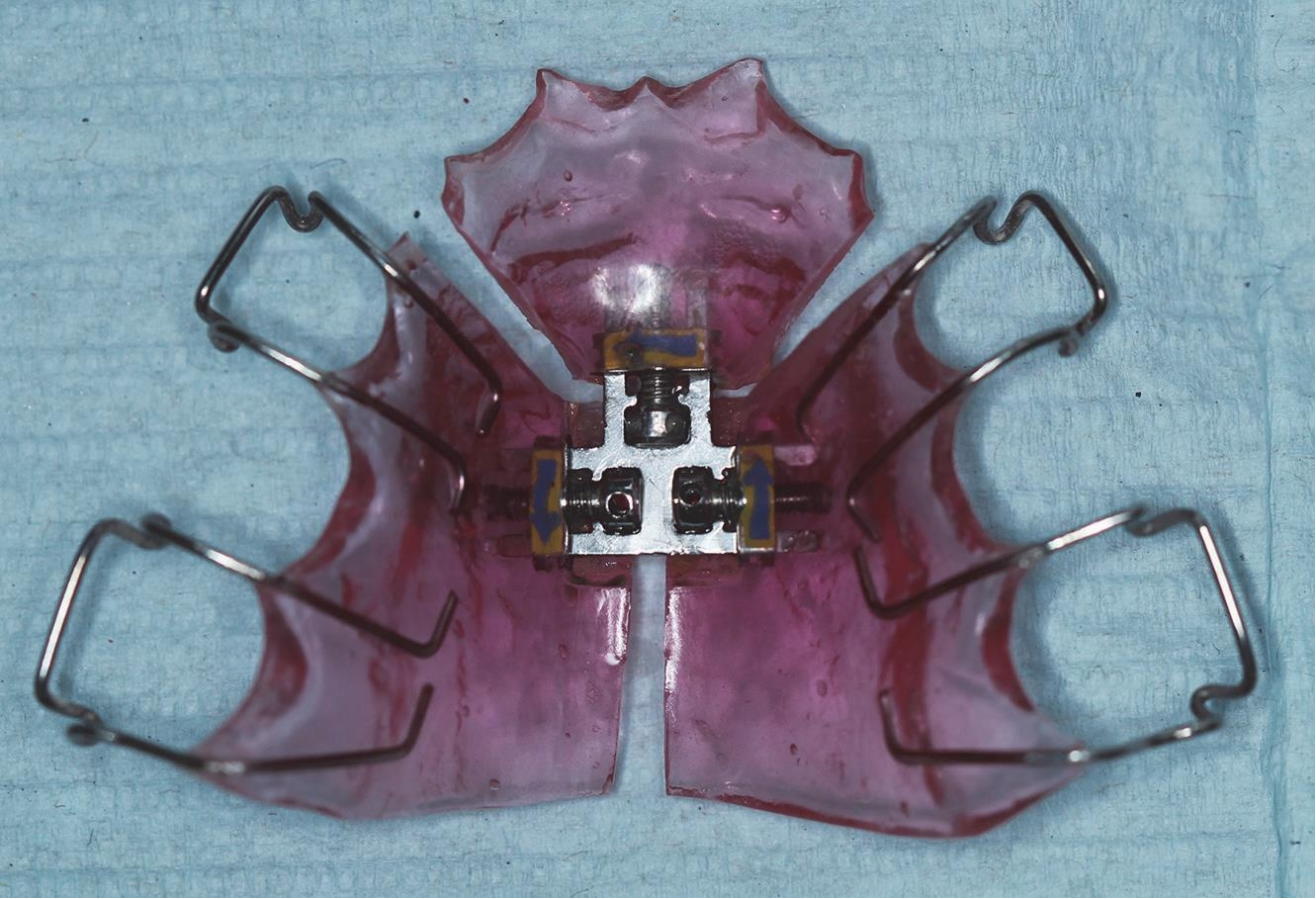
(B)

The patient was followed monthly for 12 months. Clinical outcomes were systematically documented throughout the follow‐up period. Enamel defects remained stable, with no post‐eruptive breakdown or progression. Dental hypersensitivity decreased after implementation of the preventive protocol. No new carious lesions developed. Periodontal tissues remained healthy, and no signs of infection, delayed healing, osteomyelitis, or osteonecrosis were observed following dental procedures. Slow maxillary expansion achieved a transverse increase of ~3 mm, resulting in improved arch coordination and space distribution for erupting teeth. Baseline and follow‐up occlusal photographs demonstrated improved transverse arch coordination after expansion (Figure 4A–D). Overall oral hygiene indices improved progressively, and adherence to home‐care recommendations was satisfactory.

Figure 4Occlusal views of the maxillary and mandibular dentition obtained at baseline and during follow‐up in a pediatric patient with infantile osteopetrosis. (A) Initial maxillary occlusal view. (B) Initial mandibular occlusal view. (C) Maxillary occlusal view during follow‐up showing arch coordination changes after slow maxillary expansion. (D) Mandibular occlusal view during follow‐up.

**Figure:**
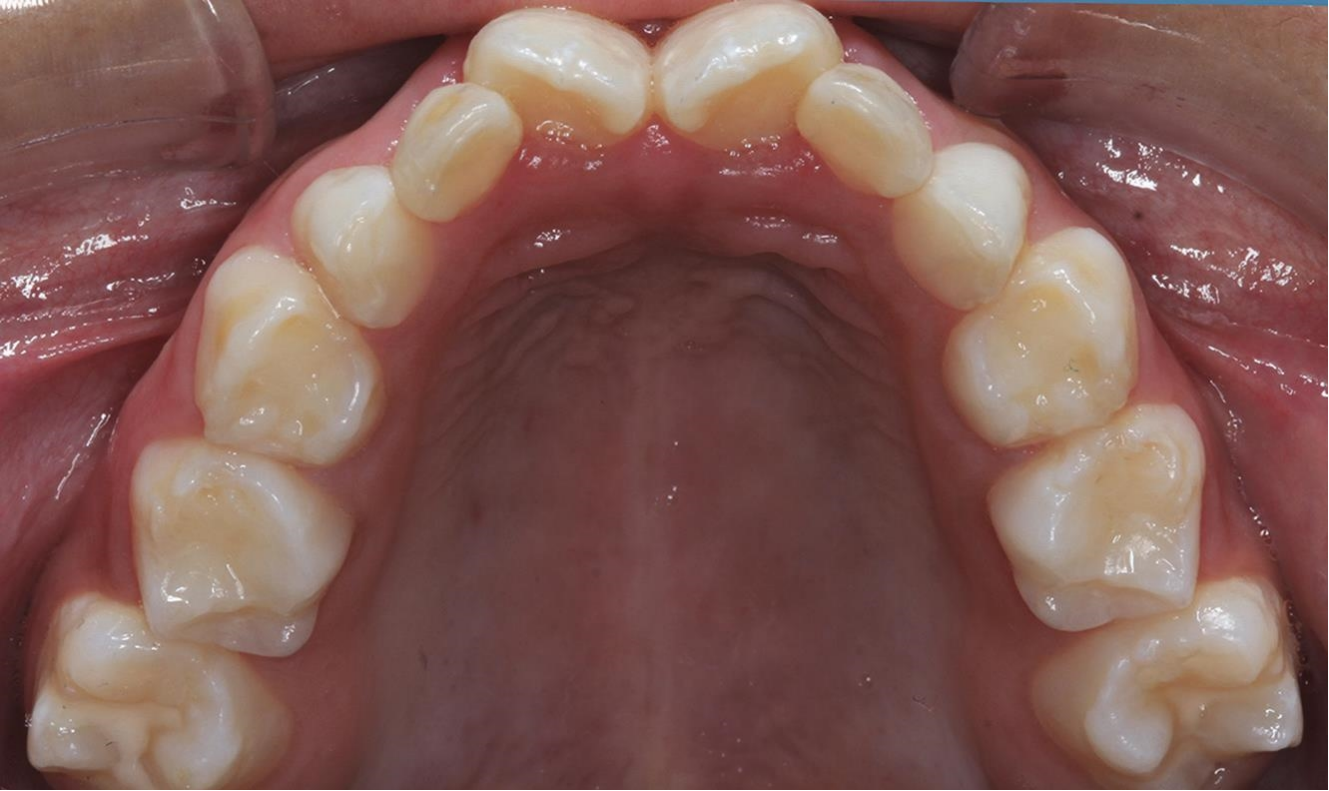
(A)

**Figure:**
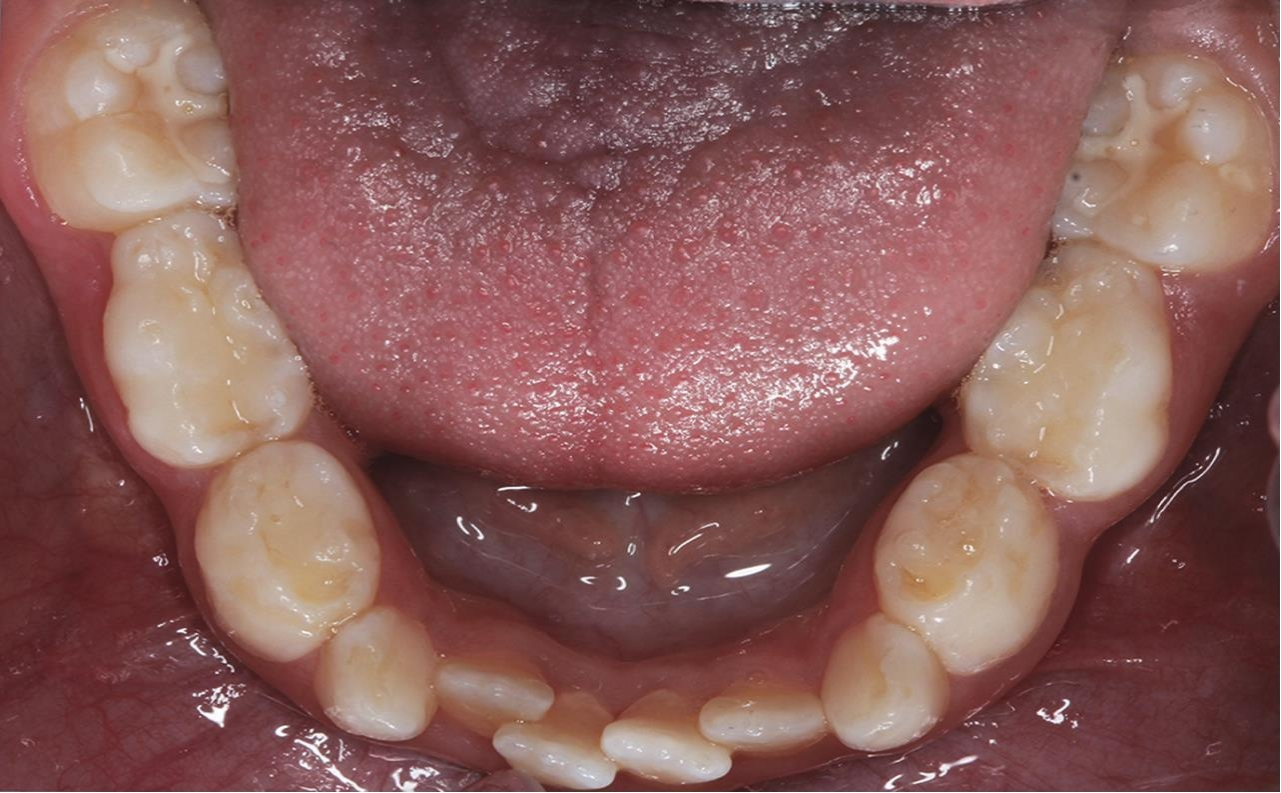
(B)

**Figure:**
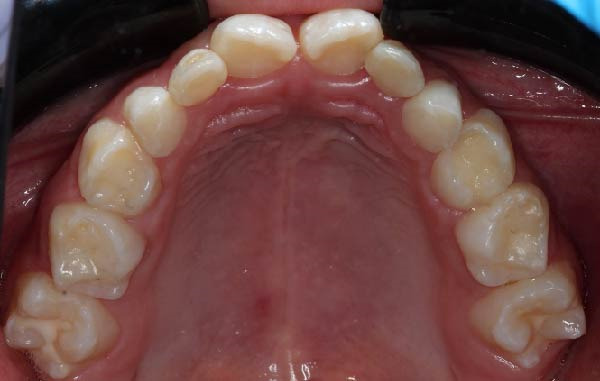
(C)

**Figure:**
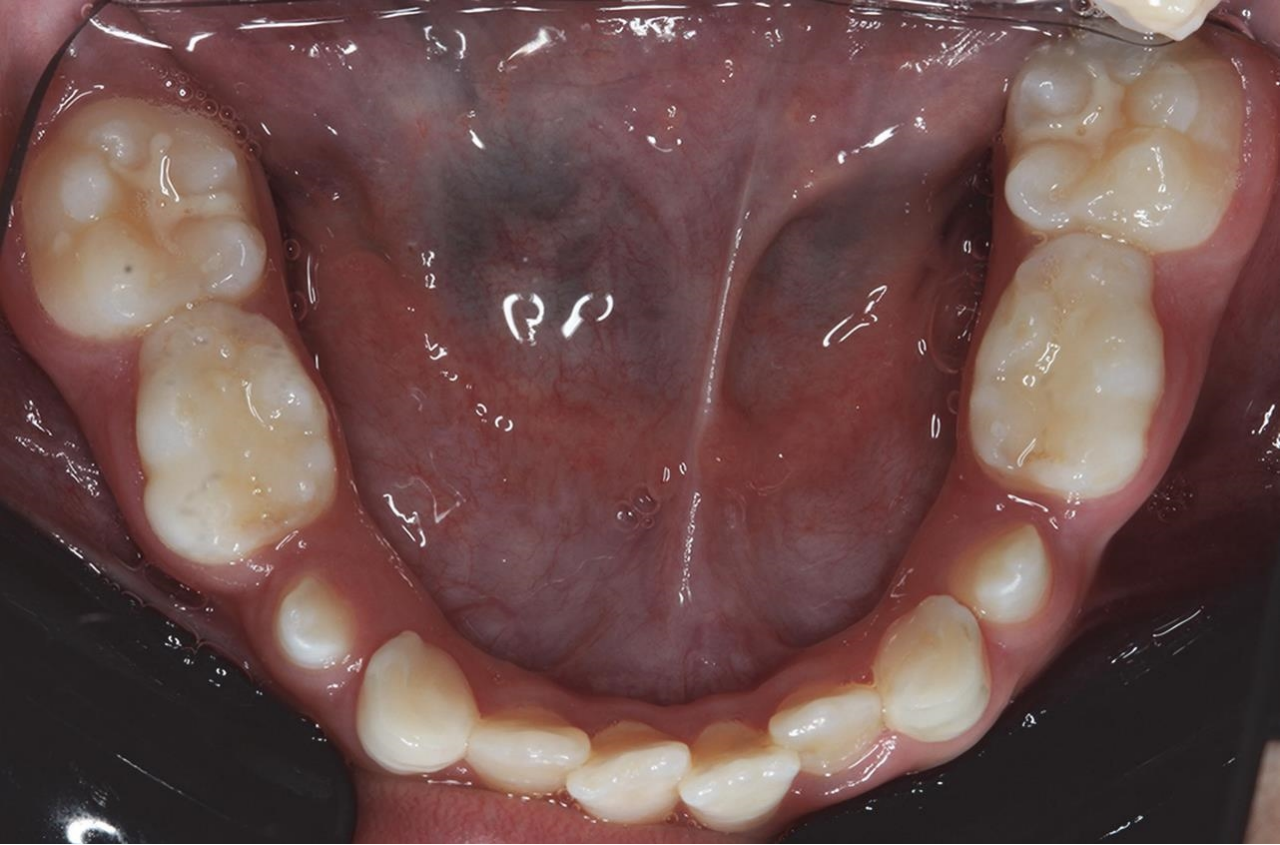
(D)

## 3. Discussion

Although osteopetrosis has been extensively described from both genetic and pathophysiological perspectives, its dental implications continue to represent a significant diagnostic and therapeutic challenge in clinical practice. The hallmark feature of the disease—abnormally increased bone density caused by osteoclast dysfunction—directly affects craniofacial growth, tooth eruption, and postoperative healing. Consequently, routine dental procedures may carry higher risks than in healthy pediatric patients [3, 9].

The three clinical forms of osteopetrosis (infantile malignant, intermediate, and adult) differ markedly in severity and prognosis, with the autosomal recessive infantile form being the most severe [1]. Despite its low global prevalence, certain populations demonstrate higher incidence rates related to hereditary and consanguinity factors, as reported in Costa Rica [3, 4]. In these settings, dental professionals may play an important role in early recognition, since oral manifestations can represent some of the earliest or most persistent signs of the disease.

Clinically, systemic complications such as anemia, skeletal deformities, and cranial nerve compression are well documented, and hematopoietic stem cell transplantation remains the treatment of choice for severe forms, significantly improving survival and quality of life [2, 8, 11]. However, even after successful transplantation, disturbances in dental and craniofacial development frequently persist. Jälevik et al. reported that children treated with bone marrow transplantation may continue to exhibit enamel defects, delayed eruption, and abnormal root formation, indicating that dental sequelae may not be fully reversible [12]. The findings observed in our patient, including oligodontia, hypoplastic enamel, and altered root morphology, are consistent with these reports and illustrate the long‐term oral consequences of early osteoclastic dysfunction.

Oral manifestations frequently described in the literature include dental agenesis, delayed or failed eruption, enamel hypoplasia and hypomineralization, and abnormalities in crown and root morphology. More severe complications, such as mandibular osteonecrosis and osteomyelitis, may occur, particularly following dental extractions or odontogenic infections [9, 13]. Taken together, these clinical features have important implications for pediatric dental practice. For clarity and clinical applicability, the principal oral manifestations, differential diagnoses, and management considerations relevant to pediatric dental practice are summarized in Table 1.

**Table 1:** Clinical considerations of infantile osteopetrosis relevant to pediatric dental practice.

Category	Description
Common oral manifestations	• Dental agenesis (oligodontia) • Enamel hypoplasia and hypomineralization • Crown and root malformations • Delayed or failed tooth eruption • Dental hypersensitivity • Mandibular osteomyelitis or osteonecrosis
Differential diagnosis	• Hypohidrotic ectodermal dysplasia • Hereditary skeletal dysplasias (e.g., cleidocranial dysplasia) • Disorders of calcium and phosphorus metabolism
Clinical implications for the pediatric dentist	• Early recognition of oral manifestations and referral for systemic evaluation • Interdisciplinary coordination with hematology, genetics, and orthodontics • Careful treatment planning and minimization of invasive procedures • Emphasis on preventive care and strict oral hygiene protocols • Regular follow‐up to monitor dental development and prevent complications

For this reason, prevention‐centered care is widely recommended. Detailleur et al. [10] emphasized that minimizing invasive procedures and prioritizing preventive strategies constitute the safest approach for these patients. In accordance with these recommendations, our management prioritized intensive oral hygiene instruction, regular professional prophylaxis, topical fluoride therapy, sealants, and dietary counseling. The absence of new carious lesions and the progressive improvement in oral hygiene during follow‐up support the effectiveness of this conservative strategy.

When extractions are unavoidable, careful planning becomes essential. Previous case reports have described severe postoperative infections and osteomyelitis requiring prolonged antibiotic therapy or surgical management [9, 14]. Therefore, in the present case, extractions were limited to teeth with poor prognosis and were performed using minimally traumatic techniques combined with strict asepsis and antibiotic prophylaxis. The absence of delayed healing or infectious complications suggests that such precautions may significantly reduce surgical risks.

Orthodontic management in osteopetrosis remains scarcely documented. Increased bone density may compromise physiological tooth movement and potentially increase the risk of complications when excessive forces are applied [3, 9]. Nevertheless, gradual and carefully controlled orthopedic approaches may offer safer alternatives. Successful completion of orthodontic therapy using conservative biomechanics has been reported in isolated case reports [15]. In our patient, slow maxillary expansion using a Bertoni appliance allowed progressive transverse development without adverse effects, demonstrating that interceptive orthodontic therapy can be safely implemented when close monitoring is adopted. Similar multidisciplinary rehabilitation strategies have been described in complex cases requiring coordinated dental and medical care [13].

Radiographic and histological findings reported in the literature, including generalized osteosclerosis, reduced medullary spaces, and impaired hematopoiesis, further explain the biological basis for the cautious clinical approach required in these patients [16]. Together, these structural characteristics justify the emphasis on prevention, minimal invasiveness, and close follow‐up adopted in the present case.

From a broader perspective, this report highlights the pivotal role of the pediatric dentist as part of an interdisciplinary healthcare team. Early identification of oral alterations, timely referral, individualized treatment planning, and long‐term monitoring are essential to prevent complications and preserve oral function. Given the rarity of osteopetrosis, well‐documented clinical reports contribute valuable practical guidance that complements existing consensus recommendations and supports evidence‐based dental management.

This report has several limitations inherent to single‐case studies. The follow‐up period was limited to 12 months, which does not allow evaluation of long‐term craniofacial development or orthodontic stability. In addition, advanced imaging techniques such as cone‐beam computed tomography were not performed, which could have provided further information regarding bone microstructure and orthodontic response in osteosclerotic bone. Future longitudinal studies are needed to better understand dental development and orthodontic outcomes in patients with osteopetrosis.

Overall, the favorable outcomes observed in this patient demonstrate that comprehensive, minimally invasive, and prevention‐oriented strategies can effectively maintain oral health and improve quality of life in children affected by osteopetrosis.

## 4. Conclusion

Given the rarity of infantile osteopetrosis, well‐documented clinical reports are essential to expand current knowledge regarding its oral manifestations and dental management. Although systemic control can be achieved through hematopoietic stem cell transplantation, significant dental sequelae may persist and require long‐term follow‐up.

This case demonstrates that a prevention‐centered and minimally invasive approach, including intensive fluoride therapy, strict plaque control, cautious surgical techniques, and gradual orthodontic biomechanics, can be safely implemented in pediatric patients with osteopetrosis. The absence of infectious or osteonecrotic complications and the favorable functional outcomes observed during follow‐up support the feasibility of conservative dental management.

Early recognition of oral signs and close interdisciplinary coordination are fundamental to preventing complications and improving oral health and quality of life in affected children.

## Author Contributions

**Maria Amalia Cruz-Morera:** conceptualization, investigation, writing – original draft. **Katherine Molina-Chaves:** investigation, data curation, visualization. **Adrian Gomez-Fernandez:** conceptualization, supervision, writing – review and editing.

## Funding

This research received no external funding.

## Ethics Statement

This clinical case was conducted in accordance with the ethical principles outlined in the Declaration of Helsinki and complies with institutional guidelines for the dissemination of clinical information for academic and educational purposes.

## Consent

Written informed consent was obtained from the patient’s parent for the publication of clinical and radiographic information, as well as written assent from the patient.

## Conflicts of Interest

The authors declare no conflicts of interest.

## Data Availability

The data that support the findings of this study are available from the corresponding author upon reasonable request.
